# Hypoxia Supports Differentiation of Terminally Exhausted CD8 T Cells

**DOI:** 10.3389/fimmu.2021.660944

**Published:** 2021-05-07

**Authors:** Nadia Bannoud, Tomás Dalotto-Moreno, Lucía Kindgard, Pablo A. García, Ada G. Blidner, Karina V. Mariño, Gabriel A. Rabinovich, Diego O. Croci

**Affiliations:** ^1^ Laboratorio de Inmunopatología, Instituto de Histología y Embriología de Mendoza (IHEM), Consejo Nacional de Investigaciones Científicas y Técnicas (CONICET), Mendoza, Argentina; ^2^ Facultad de Ciencias Médicas, Universidad Nacional de Cuyo, Mendoza, Argentina; ^3^ Laboratorio de Inmunopatología, Instituto de Biología y Medicina Experimental (IBYME), Consejo Nacional de Investigaciones Científicas y Técnicas (CONICET), Buenos Aires, Argentina; ^4^ Laboratorio de Glicómica Funcional y Molecular, Instituto de Biología y Medicina Experimental (IBYME), Consejo Nacional de Investigaciones Científicas y Técnicas (CONICET), Buenos Aires, Argentina; ^5^ Facultad de Ciencias Exactas y Naturales, Universidad de Buenos Aires, Buenos Aires, Argentina; ^6^ Facultad de Ciencias Exactas y Naturales, Universidad Nacional de Cuyo, Mendoza, Argentina

**Keywords:** Hypoxia, CD8 T cell exhaustion, immunosuppression, VEGF-A, anti cancer agents

## Abstract

Hypoxia, angiogenesis, and immunosuppression have been proposed to be interrelated events that fuel tumor progression and impair the clinical effectiveness of anti-tumor therapies. Here we present new mechanistic data highlighting the role of hypoxia in fine-tuning CD8 T cell exhaustion *in vitro*, in an attempt to reconcile seemingly opposite evidence regarding the impact of hypoxia on functional features of exhausted CD8 T cells. Focusing on the recently characterized terminally-differentiated and progenitor exhausted CD8 T cells, we found that both hypoxia and its regulated mediator, vascular endothelial growth factor (VEGF)-A, promote the differentiation of PD-1^+^ TIM-3^+^ CXCR5^+^ terminally exhausted-like CD8 T cells at the expense of PD-1^+^ TIM-3^-^ progenitor-like subsets without affecting tumor necrosis factor (TNF)-α and interferon (IFN)-γ production or granzyme B (GZMB) expression by these subpopulations. Interestingly, hypoxia accentuated the proangiogenic secretory profile in exhausted CD8 T cells. VEGF-A was the main factor differentially secreted by exhausted CD8 T cells under hypoxic conditions. In this sense, we found that VEGF-A contributes to generation of terminally exhausted CD8 T cells during *in vitro* differentiation. Altogether, our findings highlight the reciprocal regulation between hypoxia, angiogenesis, and immunosuppression, providing a rational basis to optimize synergistic combinations of antiangiogenic and immunotherapeutic strategies, with the overarching goal of improving the efficacy of these treatments.

## Introduction

In the past years, it has become increasingly clear that tumor cells alone are not sufficient to generate cancer. The tumor microenvironment (TME) (i.e.: endothelial vascular and lymphatic cells, immune cells and stromal fibroblasts, among others) are key players in tumor progression (1, 2) and play a central role in acquired resistance to targeted therapies (3, 4). In this regard, the TME has been proposed to be an attractive target for the generation of anticancer therapies including immunotherapy, antiangiogenic and targeted therapies (4, 5). Antiangiogenic therapies target the ability of cancer cells to generate an abnormal vasculature that engenders a hostile microenvironment. Strikingly, major hallmarks of this adverse scenario are hypoxia and acidic pH, which fuel immunosuppression and promote impairment of effector T-cell function (6, 7). In this scenario, tumor hypoxia emerges as a major driving force that influences not only malignant cells but also the TME, impairing effector immune responses and promoting angiogenesis, by affecting cell migration and endothelial cell adhesion or directly influencing immune cell differentiation and function (8, 9). In this sense, immunomodulatory molecules such as vascular endothelial growth factor (VEGF-A), hepatocyte growth factor (HGF), angiopoietins, adenosine, transforming growth factor-β (TGF-β), and galectins (9–11) are key soluble mediators that link these pro-tumoral functions (12).

Despite significant progress in understanding the molecular components of hypoxia-regulated programs in the TME (13), the cellular mechanisms and mediators coupling tumor hypoxia and CD8 T cell exhaustion remain elusive. Although the programs that govern T cell exhaustion are still under debate, there is a consensus that it comprises phenotypically and functionally heterogeneous exhausted CD8 (exhCD8) T cells. In this perspective article, we discuss the role of hypoxia in fine-tuning CD8 T-cell exhaustion, in an attempt to reconcile previous studies and emerging evidence describing functional features of newly characterized terminal and progenitor features of newly characterized terminal and progenitor exhCD8 T cells.

## Hypoxia in the Promotion of Immune Tolerance

Tumor hypoxia impairs immune responses by influencing mechanisms that bribe immune cells to become immunosuppressive (14). These molecular pathways include the hypoxia inducible factor-1 alpha (HIF-1α)-dependent induction and recruitment of Foxp3^+^ regulatory T cells (Tregs) through mechanisms involving TGF-β-driven STAT-3 signaling (15, 16), the release of CCL28 chemokine by tumor cells (17) and CCL22 by tumor-associated macrophages (TAMs) (18). Moreover, T cell receptor (TCR) cross-linking in HIF-1α-deficient T cells skews the balance towards a pro-inflammatory cytokine profile (19), suggesting that HIF-1α may function as a negative regulator of T-cell differentiation and cytokine production.

Hypoxia has also been associated with dysregulated activity of tumor-associated myeloid cells (20–22). Differentiation, recruitment, and polarization of TAMs may be regulated by tumor cells *via* VEGF-A, HIF-1α and CCL2-dependent mechanisms (23, 24). Tumor cell expression of VEGF-A contributes to recruitment of Tregs to the TME (25), promotes CD8 T cell exhaustion (26), and impairs dendritic cell (DC) maturation (27). Furthermore, under hypoxic conditions HIF-1α, but not HIF-2α activation contributes to up-regulation of PD-L1 in myeloid-derived suppressor cells (MDSCs), TAMs, and DCs, endowing these cells with tolerogenic activity (28–30). These data suggest that simultaneous blockade of HIF-1α and immune checkpoints such as programmed death-1 (PD-1) and cytotoxic T-lymphocyte antigen-4 (CTLA-4) could represent a novel approach for combinatorial cancer immunotherapy.

Despite the well-established proangiogenic and anti-inflammatory roles of hypoxia through the stabilization of HIF-α proteins, more recent studies revealed that these transcription factors contribute to inflammation by promoting Th17 cell differentiation (31, 32). Additionally, prolyl hydroxylase proteins (PHD), together with *vhl* genes, induce O_2_-tagged-dependent HIF-1α degradation; this effect restrains the function of CD4 and CD8 T cells and increases Treg cell expansion in lung tissues, thereby promoting a permissive niche for lung metastasis (33, 34). These data highlight the need of further exploration of the interplay between hypoxia and inflammation in the TME. In this complex scenario, the impact of hypoxia in CD8 T cell immunoregulation is not fully understood.

## CD8 T Cell Exhaustion

Exhausted T cells were initially described as hyporesponsive or hypofunctional effector T cells characterized by sustained expression of multiple inhibitory receptors, progressive loss of effector functions (cytotoxicity and cytokine production), reduced proliferative capacity, altered expression and function of key transcription factors and dysregulation of epigenetic programs. Even though these phenotypic features have been widely used as hallmarks of T-cell exhaustion programs, enabling the distinction of naive (Tn), effector (T eff) and memory T cells (Tm) (35, 36), recent transcriptional and epigenetic studies have demonstrated that exhaustion is not merely a transient impairment of the functionality of T cells. Instead, T-cell exhaustion involves distinct states of T-cell differentiation with a continuum of phenotypic and functional intermediate states (37, 38). Thus, a deeper understanding of the factors that control exhaustion programs is central for shaping the course of chronic infections and cancer.

During an acute immune response, immune receptors are transiently expressed by Tef cells to limit immunopathology and autoimmunity (39, 40). However, in chronic infections and cancer, sustained expression of immune checkpoint molecules gives rise to the expansion of exhausted T cells. Among these co-inhibitory molecules, CTLA-4 (CD152), PD-1 (CD279), T cell immunoglobulin domain and mucin domain-containing protein 3 (TIM-3/HAVCR2/CD366), lymphocyte activation gene-3 (LAG-3/CD223), T cell immunoreceptor with Ig and ITIM domains (TIGIT), B and T lymphocyte attenuator (BTLA/CD272), 2B4 (CD244) and CD160 (41–43), play key roles in T-cell exhaustion and represent important targets for the design of new generation anticancer immunotherapies (42).

Although different signals may promote CD8 T-cell exhaustion, persistent antigen stimulation appears to be the major driving force leading to a T-cell exhausted phenotype (37, 44). Impairment of CD8 T-cell functionality is favored when CD4 T-cell function is affected by diminished IL-21 production (45, 46). Moreover, increased levels of pro-inflammatory cytokines, such as type I interferons (IFNs) and IL-6, or immunosuppressive cytokines including IL-10 and TGF-β1 (45, 47) contribute to shape an exhausted phenotype. In addition, microenvironmental factors, such as hypoxia and nutrient deprivation (e.g., glucose, amino acids, glutamine), can limit T-cell activity and consequently impair the immune response by modulating metabolic pathways (42, 48, 49). Thus, T-cell exhaustion represents an evolutionary adaptation to conditions of chronic antigen stimulation and inflammation (38), favoring tissue repair following an inflammatory injury.

**Figure 1:**
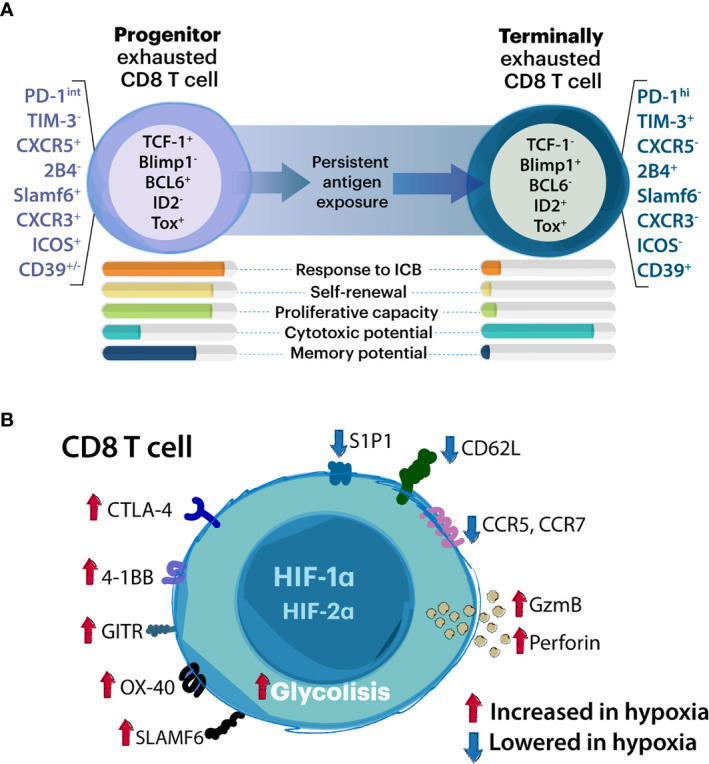
Association between CD8 T cell exhaustion and hypoxia. **(A)** Schematic representation of progenitor and terminally exhausted T cell subsets. Although T cell exhaustion comprises a wide range of exhausted states, two major subsets of exhCD8 T cells have been studied in detail: the progenitor and the terminally exhausted T cell population. Whereas progenitor exhausted T cells exhibit proliferative potential, and stemness properties and can be rescued by immune checkpoint blockade (ICB) therapies, terminally exhausted T cells have higher cytotoxic potential but represent a terminal differentiation state and cannot be rescued by ICB. Proposed key molecules which discriminate these subpopulations are listed. **(B)** Modulation of CD8 T cell functions by hypoxia. Through HIF-1α and HIF2α- dependent mechanisms, hypoxic stimuli favor glycolytic anaerobic metabolism promoting T cell receptor (TCR) signaling. These include enhanced perforin and granzyme-B (GzmB) release as well as expression of immune checkpoint molecules (including both activators and inhibitors). Hypoxia inhibits expression of chemokine and cytokine receptors and adhesion molecules.

Recently, high-dimensional studies identified approximately nine phenotypic subtypes of exhausted T cells (50), but to date two major subsets of exhCD8 T cells have been described, namely progenitor or stem-like subset, and terminally-exhausted populations (**Figure 1A**) (36, 38). The identification and characterization of these exhCD8 T cell subpopulations represents a paradigm shift in the conception of cytotoxic T cells during the course of antitumor responses. Notably, the balance between progenitor and terminally exhCD8 T cells determines the cytotoxic potential and longevity required for mounting an effective immune response (41).

The so called exhCD8 T cells progenitor population can be defined as PD-1^int^ (36) CXCR5^+^ (51) or Slamf6^+^/Ly108 (38, 52) lacking expression of TIM-3 (51), while terminally exhCD8 T cells are identified as PD-1^hi^ (36, 37) or TIM-3^+^ (37, 51). Although several transcription factors have been proposed as key determinants of exhaustion programs, recent studies highlighted a dynamic interplay between T Cell Factor-1 (TCF-1) and thymocyte selection-associated high mobility group box protein (TOX), in the control of different exhausted populations (51, 53–56). TCF-1-expressing progenitor T cells are also characterized by enhanced proliferative capacity, polyfunctional cytokine production, and long-term persistence in the absence of antigen. Two progenitor interchangeable states have been described with functional and anatomical differences, but similar epigenetic programming that can be catalogued in the lymphoid tissue resident Texh Prog1 and the blood accessible Texh Prog2, which eventually give rise to terminally exh T cells inside the tumor tissue or inflammatory site (53). The terminally exhCD8 T cells are characterized by coexpression of B lymphocyte-induced maturation protein-1 (Blimp-1) (56) and effector genes (e.g. GZMB), which sustain their cytotoxic activity, and exhibit reduced long-term survival and polyfunctional cytokine production (41). The main implications of the two subsets of CD8 T cells in TME rely on their potential to respond to PD-1 immune checkpoint blockade (ICB); the progenitor population expands giving rise to terminally-differentiated exhausted subsets (38, 51), a primary cytotoxic CD8 T cell population in the TME. Therefore, a balance of both progenitor and terminally-exhausted populations may be required for effective control of tumors and chronic infections (36, 38). With the major goal of improving therapeutic interventions, new studies are required to explore the relevance of these phenotypical changes in pathophysiological settings and their crosstalk with environmental factors in inflammatory and TME.

## Hypoxia Modulates CD8 T Cell Differentiation

CD8 T cell reinvigoration and its relevance in immunotherapy have been extensively discussed (41, 57). However, the role of hypoxia in shaping the phenotype of these cells is a matter of debate. In tumors, adipocyte tissue, and secondary lymphoid organs, CD8 T cells are preferentially localized in hypoxic zones (58, 59). In pioneering studies, hypoxia was reported as a critical factor that potentiates CD8 T cells lytic properties but interrupts their development (60, 61) (**Figure 1B**). In this sense, VHL-mediated HIF-1α and HIF-2α stabilization blunts the differentiation of CD8 T cells *in vitro* but increases GZMB expression, promoting the capacity of these cells to control tumor growth and persistent viral infections (34). Additionally, GZMB and the activation-associated costimulatory receptors 4-1BB, GITR, and OX40 were found to be increased in CD8 T cells exposed to hypoxia (62). In this sense, HIF-1α but not HIF-2α drives CD8 T cell migration and effector function (63). However, the expression of key immune checkpoint receptors such as LAG-3 and CTLA-4 was also increased in these CD8 T cells under hypoxia in a VHL/HIF-1α-dependent mechanism (34). In addition, hypoxia fuels T cell exhaustion through a miRNA-24-dependent MYC dysregulation that affects mitochondrial function and metabolism (64), suggesting a complex regulatory program driven by hypoxia in CD8 T cells.

Interestingly, the relevance of hypoxic regions in the tumor tissue varies from acute hypoxia surrounding blood vessels that slowly consolidates in chronic hypoxia according to oxygen supply. The interplay between distinct hypoxic stages may determine local T cell cytotoxicity and global success of a wide variety of therapies (65). On the other hand, residence of TCF1^+^ CD8 progenitor T cells appear to depend on MHCII^hi^ DC niches that ensure their survival and further conversion to a differentiated late exhausted and highly cytotoxic progeny. Notably, these tertiary lymphoid structures correlate with lymphatic and blood vessel infiltration into the tumor (66). In conclusion, these data suggest a spatial correlation between immunosuppression, hypoxia and terminally-differentiated exhausted T cells.

Under hypoxia, the primary biological mediator of HIF activation is VEGF-A (67). In addition to its functions as a regulator of the angiogenic process (68), this growth factor promotes a dysfunctional phenotype in CD8 T cells by increasing the expression of co-inhibitory molecules including PD-1, CTLA-4, and TIM-3 (26, 63). Moreover, VEGF-A upregulation in microsatellite stable colorectal cancers promotes resistance to anti-PD-1 immunotherapy by antagonizing the effector function of CD8 T cells and favoring expansion of exhCD8 T cells *via* induction of a TOX-mediated transcriptional program (69). Under hypoxic conditions, CD8 T cells maintain or even increase their cytotoxic capacity but slow down their development, secrete less IFN-γ and IL-2, and enhance the expression of immune inhibitory checkpoints (58, 69). However, evidence linking hypoxia to exhCD8 T cell differentiation is still scarce. To fill this gap and reconcile the apparently controversial data, we conducted *in vitro* experiments exploring how hypoxia influences generation and functionality of different subsets of exhCD8 T cells.

## Hypoxia and VEGF-A Favor Differentiation to Terminally Exhausted CD8 T Cells

**Figure 2:**
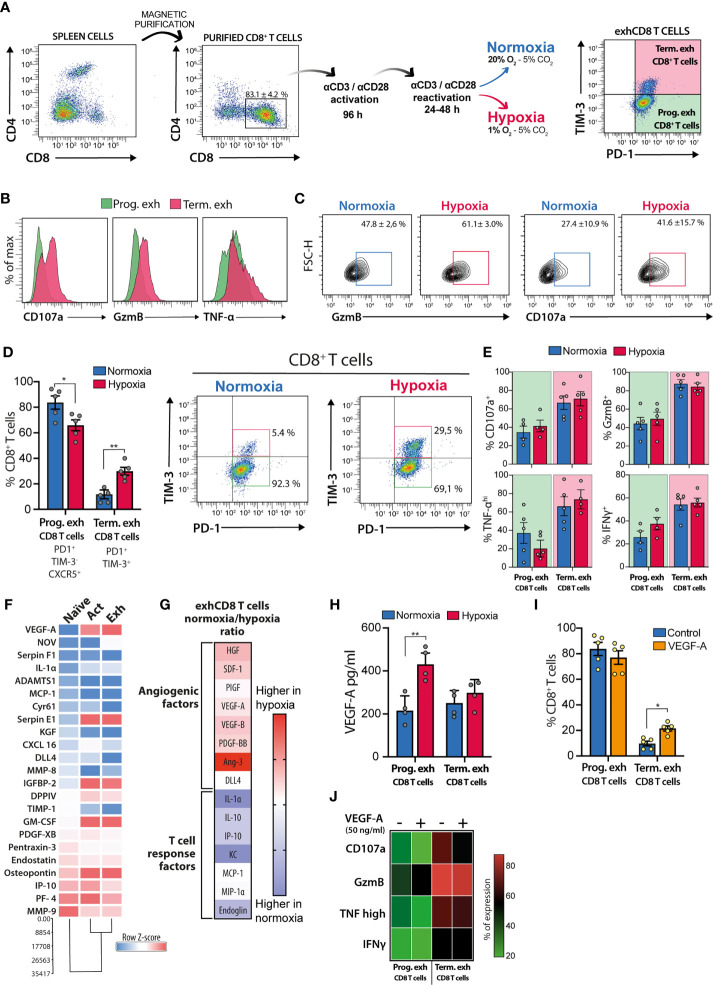
Hypoxia and VEGF-A promote differentiation of terminally exhCD8 T cells. **(A)** Schematic representation of workflow and gating strategy for *in vitro* differentiation of exhCD8 T cells. **(B)** Representative histograms showing intracellular staining of CD107a, Granzyme-B (GzmB), and TNF-α in progenitor (green) or terminally (pink) exhCD8 T cells. **(C)** Representative contour plots showing GzmB and CD107a expression in CD8^+^ PD-1^+^ T cells after 24 h exposure to hypoxia (1% O_2_) or normoxia (20% O_2_). **(D)** Differentiation of PD-1^+^TIM-3^-^CXCR5^+^ progenitor exhCD8 T cells and PD-1^+^TIM-3^+^ terminally exhCD8 T cells under normoxic (blue bars) or hypoxic (red bars) conditions. Left, percentage of each subpopulation. Right, representative density plots showing the subpopulations. Data are the mean ± SEM of five independent experiments. **(E)** Determination of cytokine expression by intracellular flow cytometry in different exhCD8 T cell subpopulations under normoxic or hypoxic conditions. Data are the mean ± SEM of 4 independent experiments. **(F)** Heat map representing normalized row Z-score of semiquantitative cytokine array analysis of angiogenic factors secreted by CD8^+^ T cells during the differentiation process. Data shows densitometric determinations and cluster analysis of pooled supernatants from five independent experiments. **(G)** Representation of the ratio of cytokines secreted by exhCD8 T cells under normoxic or hypoxic conditions. Heat maps represent the ratio of normalized densitometric data for each cytokine in normoxia versus hypoxia. **(H)** VEGF secretion by PD-1^+^TIM-3^-^CXCR5^+^ progenitor exhCD8 T cells and PD-1^+^TIM-3^+^ terminally exhCD8 T cells under normoxic (blue bars) or hypoxic (red bars) conditions. Data are the mean ± SEM of four independent experiments. **(I)** Differentiation of PD-1^+^TIM-3^-^CXCR5^+^ progenitor exhCD8 T cells and PD-1^+^TIM-3^+^ terminally exhCD8 T cells in the presence (orange bars) or in the absence (blue bars) of VEGF-A (50 ng/ml). Data are the mean ± SEM of five independent experiments. **(J)** Heat map representation of intracellular cytokines determined by flow cytometry in different exhCD8 T cells subpopulations in the presence or the absence of VEGF-A (50 ng/ml). Each row represents the mean ± SEM of the percentage of cells expressing each cytokine in four independent experiments. *p < 0.05, **p < 0.01.

In order to address the role of hypoxia in exhCD8 T cell differentiation, we first performed *in vitro* experiments (**Figure 2A**) exposing differentiated exhCD8 T cells to hypoxic or normoxic conditions, and explored the frequency and functionality of the different exhCD8 T cells subsets (**Figures 2B–D**). According to previous reports, *in vitro* differentiation of CD8^+^ T cells resulted in an exhausted phenotype characterized by expression of PD-1 and TIM-3 co-inhibitory receptors (63, 69). Among them, two subpopulations can be clearly distinguishable as PD1^+^TIM3^-^ and PD1^+^TIM3^+^ (**Figure 2A**). While the former displayed a phenotype characterized by lower cytotoxic potential, demonstrated by GzmB expression and CD107a mobilization, the latter (PD1^+^TIM3^+^) subpopulation exhibited an increased cytotoxic potential (**Figure 2B**), with higher expression of TNF-α and IFN-γ, compared with the PD1^+^TIM3^-^ cells (**Figure 2B**). These features are consistent with the two well-characterized exhCD8 T populations recognized so far, the progenitor and the terminally exhausted subsets (37). In this sense, and as previously reported, hypoxic conditions enhanced the cytotoxic profile of CD8^+^ T cells, assessed by GzmB expression and CD107a mobilization to the cell surface (**Figure 2C**). Notably, when we further analyzed CD8 T subpopulations, we found that hypoxia increased the percentage of terminally exhausted-like cells at the expense of the progenitor-like subset (**Figure 2D**). We confirmed that PD1^+^TIM3^-^ cells expressed CXCR5^+^, a typical marker of the progenitor exhausted phenotype (51), while PD1^+^TIM3^+^ cells displayed a terminally exhausted-like phenotype (**Figure 2D**). Nonetheless, no differences in cytokine production were observed in these populations under hypoxic conditions (**Figure 2E**), suggesting that hypoxia favors the generation of a terminally exhausted phenotype in CD8 T cells with no evident changes in their cytokine secretion profile.

To study whether hypoxia could affect secretion of pro-angiogenic factors, we performed an angiogenic cytokine array to assess cytokines expressed by naïve, activated, and exhCD8 T cells, under normoxic or hypoxic conditions. Interestingly, activation of CD8^+^ T cells imprinted these cells with a predominant proangiogenic phenotype, characterized by greater secretion of soluble proangiogenic factors (**Figure 2F**). Moreover, hypoxia-driven differentiation of terminally exhausted T cells resulted in enhanced expression of pro-angiogenic mediators when compared with exhCD8 T cells exposed to normoxic conditions (**Figure 2G**). Under hypoxic conditions, differentiated exhCD8 T cells displayed a broad pro-angiogenic program characterized by higher levels of pro-angiogenic molecules such as VEGF-A, platelet-derived growth factor (PDGF), placental growth factor (PIGF), and angiopoietin-3 (Ang3). On the other hand, under normoxic conditions, exhCD8 T cells showed a secretome associated with regulation of T cell responses, as shown by IL-10 and the cell surface receptor endoglin, a component of the TGF-β-signaling receptor family (**Figure 2G**). Interestingly, hypoxia selectively increased VEGF-A secretion in the progenitor exhCD8 T cell subset (**Figure 2H**).

Finally, given the role of VEGF-A in promoting CD8 T cell exhaustion (26) and its regulated expression in exhCD8 T cell subsets differentiation *in vitro* (**Figures 2F–H**), we explored its effects in the frequency of progenitor and terminally exhCD8 T cell subsets. Remarkably, VEGF-A (50 ng/ml) favored the differentiation of CD8^+^ T cells toward a terminally exhausted-like phenotype (**Figure 2I**), although at a lesser extent than hypoxia. Of note, VEGF-A did not alter the percentage of cells expressing CD107a, GzmB, TNF-α, or IFN-γ among the different exhCD8 T cell populations (**Figure 2J**). Thus, increased frequency of terminally-exhausted-like cells arising following exposure to hypoxic conditions, could be attributed, at least in part, to the effects of VEGF-A.

## Methods

Splenic CD8^+^ T cells were obtained from C57BL/6 mice bred at the animal facility of Instituto de Histología y Embriología de Mendoza (IHEM) according to NIH Guidelines for the Care and Use of Animals. All procedures were approved by the Institutional Animal Care and Use Committee of the School of Medical Science, Universidad Nacional de Cuyo (Protocol approval N° 111/2017). CD8^+^ T cells were isolated using the Dynabeads Untouched Mouse CD8 cells kit (Invitrogen) and subsequently seeded (5 x 10^5^ cells/mL) in RPMI supplemented with 10% fetal bovine serum (FBS) (Gibco) and 5.5 x 10^-5^ M 2-mercaptoethanol, in 48-well culture plates. *In vitro* stimulation was performed with immobilized anti-CD3 antibody (2.5 µg/mL; Clone BD Biosciences) and anti-CD28 antibody (2 µg/mL; Clone BD Biosciences). After 96 h, cells were re-stimulated with anti-CD3 (1.5 µg/mL) and anti-CD28 (1 µg/mL) antibodies in 10% FBS RPMI. CD8^+^ T cells were incubated under hypoxia in a modular incubator chamber (Billups-Rothenberg, San Diego, CA, USA), flushed at 2 psi for 10 min with a mixture of 1% O_2_, 5% CO_2_, and 94% N_2_. The chamber was sealed and placed in a 37°C incubator for 48 h. Control CD8^+^ T cells were placed in the same incubator at 20% O_2_. Cell cultures in both conditions were then incubated with VEGF-A (50 ng/ml, R&D systems) for 24 h at 37°C. The Zombie Green Fixable Viability Kit (Biolegend, San Diego, CA, USA) was used to exclude dead cells. CD8^+^ T cells were phenotyped by cell surface staining with anti-CD4 (clone GK1.5), anti-CD8 (clone 53-6.7), anti-TIM-3 (clone RMT3-23) anti-PD1 (clone RMP1-14) and anti-CXCR5 (clone L138D7) antibodies (all from Biolegend) in 1% BSA in PBS for 20 min at 4°C. For intracellular staining, cells were permeabilized with BD Perm/Wash buffer (BD Biosciences) and further stained with anti-Gzm-B (clone 6B11; e-biosciences) and anti-TNF-α (clone MP6-XT22), anti-IFN-*γ* (clone XMG1.2) and anti-CD107a (clone 1D4B), all from BD Pharmigen. To assess cytokine production, CD8^+^ T cells were incubated with PMA (50 ng/mL, Sigma), ionomycin (1 µg/mL, Sigma) and monensin (Golgi STOP, BD Biosciences) at 37°C. Cells were harvested after 4h and intracellular cytokines were evaluated by flow cytometry. Experiments were performed in a BD Accuri C6 Plus flow cytometer (BD Biosciences) and data was analyzed with FlowJo V10.7.1 software.

Cytokine arrays were performed with the Proteome Profiler Mouse XL Cytokine Array (R&D Systems) following manufacturer’s instructions using conditioned media from CD8^+^ T cells obtained after the differentiation process and exposed to hypoxic or normoxic conditions. Murine VEGF-A secretion was determined by ELISA (R&D systems) in conditioned media from previously sorted progenitor or terminally exhCD8 T cells.

Statistical analysis and data representation was performed using GraphPad Prism 8.2.1 Software (GraphPad, CA, USA). Student’s *t* test was used for unpaired data. Two-way ANOVA and Dunnett’s or Tukey post-tests were used for multiple comparisons. Cluster differentiation analysis from normalized row Z-score values was performed with Infostat software. *P* values of 0.05 or less were considered significant. Exact *P* values are reported in all figures.

## Discussion

In the past years, combination therapies have changed the landscape of cancer treatment (70). Interestingly, combinations of antiangiogenic therapies and ICB are currently being evaluated in several tumor types (9, 70, 71). Hypoxia, the primary driving force responsible of triggering vascularization programs, has come into the spotlight because of its concomitant immunosuppressive activity in the TME (72, 73). Hypoxia promotes T cell activation and cytotoxic activities and favors the development of exhaustion programs (4). Although there are no studies focused on the role of hypoxia or VEGF-A on different exhCD8 T cell subpopulations, it is known that expression of TIM-3, a signature of terminally-exhausted T cells, is highly up-regulated under hypoxic conditions (26, 63, 74).

Here, we showed that hypoxia promotes the differentiation of PD-1^+^TIM-3^+^ terminally exhCD8 T cells at the expense of the PD-1^+^TIM-3^-^CXCR5^+^ progenitor-like population. Although these cells exhibit a highly cytotoxic-like phenotype (38, 74), they are resistant to ICB therapies. In fact, it has been recently proposed that anti-PD1 antibodies targets the progenitor exhausted TILs, but not the terminally-exhausted T cells (38, 75). Thus, favoring expansion of the progenitor exhCD8 T cells might improve responses to ICB.

In this sense, Voron and colleagues demonstrated that VEGF-A increases the percentages of TIM-3^+-^expressing CD8^+^ T cells and highlighted the role of VEGF-A in resistance to anti-PD-1 treatment in a murine model of colorectal cancer (26). Then, Palazon and colleagues confirmed and expanded these findings suggesting a direct role of HIF-1α as an essential regulator of T cell effector responses in the TME through mechanisms involving VEGF-A regulation, angiogenesis, and T cell migration (63). In this sense, we found an up-regulation of pro-angiogenic factors upon T cell activation. Furthermore, in exhCD8 T cells hypoxia imprints a pro-angiogenic program characterized by up-regulation of Ang-3, PDGF, hepatocyte growth factor (HGF), and VEGF-A. In this regard, our study demonstrates that VEGF-A promotes CD8 T cell exhaustion by increasing the frequency of terminally-exhausted T cells. Moreover, our results, albeit limited to *in vitro* experiments, suggest a cross-talk between vascular and immune cell programs which target exhausted T cells and simultaneously foster immune escape and neovascularization. These results support the rationale of improving combinatorial therapies using HIF inhibitors or antiangiogenic therapies plus ICB in highly hypoxic tumors (70).

Although further studies are needed to understand how hypoxia links immune tolerance and angiogenesis and sustains resistance to anti-tumor therapies, studies in clear cell renal cell carcinoma (ccRCC) could give a preliminary insight into this possibility. This tumor is characterized by a high CD8^+^ T cell infiltration and loss of the tumor suppressor von Hippel-Lindau (VHL), which promotes HIF stabilization leading to activation of several oxygen-independent hypoxic transcriptional programs. In ccRCC tumors, CD8 T cell infiltration rate is typically associated with poor prognosis (76), imposing hurdles to most immunotherapeutic modalities (77). In this sense, and in line with our hypothesis, Siska and colleagues reported that phenotypical and functional differences of CD8 T cells from ccRCC involve constitutive activation of HIF-1α, which promotes an altered metabolism (78). These results shed light on the complex relationships between HIF activation and CD8 T cell functionality in the TME.

In conclusion, our findings suggest that hypoxic programs may represent an attractive target to attenuate T cell exhaustion and immunosuppression in the TME. However, further studies *in vivo* are required to examine the role of hypoxia, its cellular mediators and signaling pathways in supporting tumor-immune escape and T cell exhaustion in antigen-specific and pathologic settings. Further investigation should be aimed at exploring the intimate link between hypoxia, immunosuppression, and angiogenesis with the ultimate goal of designing more effective combinatorial modalities for treating cancer patients.

## Data Availability Statement

The raw data supporting the conclusions of this article will be made available by the authors, without undue reservation.

## Ethics Statement

All procedures were approved by the Institutional Animal Care and Use Committee of the School of Medical Sciences, Universidad Nacional de Cuyo. Approval Nº 111/2017.

## Author Contributions

NB acquired, analyzed, and interpreted data, supervised the study, and wrote the manuscript. TD-M interpreted data and revised the manuscript. LK acquired, analyzed, and interpreted data, PG analyzed and interpreted data, and revised the manuscript. AB interpreted data and revised the manuscript. KM interpreted data and revised the manuscript. GR conceived and supervised the study and wrote the manuscript. DC conceived, designed, and supervised the study, interpreted data, and wrote the manuscript. All authors contributed to the article and approved the submitted version.

## Funding

This work was supported by grants from the Argentinean Agency for Promotion of Science and Technology PICT 2016-0205 to DC and PICT 2017-0494 to GR, and Secretaría de Investigación, Relaciones Internacionales y Posgrado, SIIP-UNCuyo J069 to NB and DC. This work was also supported by the NIH grants U54 CA 0221208 to DC and GR.

## Conflict of Interest

The authors declare that the research was conducted in the absence of any commercial or financial relationships that could be construed as a potential conflict of interest.
